# Emerging Trends and Research Frontiers in Climate Change and Asthma: Insights From a Two‐Decade Bibliometric Analysis

**DOI:** 10.1155/carj/5546333

**Published:** 2026-06-22

**Authors:** Yixin Wang, Huanxia Qu, Xinchen Shu, Qi Liu, Jiaxin Cao, Yuxin Liu, Feng Jiang, Jin Shu

**Affiliations:** ^1^ Department of Pediatrics, The Fourth Affiliated Hospital of Jiangsu University, Zhenjiang, China, jdfy.cn; ^2^ Department of Blood Transfusion, Zhenjiang First People’s Hospital, Zhenjiang, China; ^3^ School of Mathematics, University of Birmingham, Birmingham, UK, birmingham.ac.uk; ^4^ Diagnostics and Therapeutics of Intractable Diseases, Intractable Disease Research Center, Graduate School of Medicine, Juntendo University, Tokyo, Japan, juntendo.ac.jp; ^5^ Department of Neonatology, Shanghai Key Lab of Reproduction and Development, Shanghai Key Lab of Female Reproductive Endocrine Related Diseases, Obstetrics & Gynecology Hospital of Fudan University, Shanghai, China

**Keywords:** asthma, bibliometric analysis, climate change, extreme weather, public health

## Abstract

**Background:**

Climate change has emerged as a major public health challenge, with significant implications for respiratory diseases such as asthma. Environmental factors, including air pollution, aeroallergens, and extreme weather events, are closely linked to asthma exacerbations, yet the global research landscape on this topic remains fragmented. This study aimed to systematically map research trends, hotspots, and emerging frontiers in the field of climate change and asthma over the past two decades.

**Methods:**

Publications from January 1, 2005, to March 31, 2025, were retrieved from the Web of Science Core Collection (WoSCC) using a defined search strategy. Only articles and reviews in English were included, resulting in a final dataset of 1033 publications. CiteSpace was employed for bibliometric analysis, including coauthorship, cocitation, and keyword co‐occurrence mapping, as well as cluster and burst detection, to identify influential contributors, collaborative networks, and thematic evolution.

**Results:**

Annual publications increased steadily, peaking in 2024. The United States and China were the most prolific countries, with Harvard T.H. Chan School of Public Health, Harvard University, and Columbia University among the leading institutions. Key research hotspots included climate‐related environmental exposures (e.g., PM2.5, ozone, and temperature variability), allergen dynamics (e.g., pollen, ragweed, and fungal spores), vulnerable populations (particularly children), and public health impacts. Emerging frontiers encompassed interdisciplinary integration, predictive modeling, mechanistic studies focusing on oxidative stress and immune regulation, and climate adaptation strategies. Despite progress, challenges remain in integrating asthma‐specific measures into climate–health policies, conducting long‐term multinational studies, and advancing translational research.

**Conclusions:**

This bibliometric analysis provides a comprehensive overview of the global research landscape on climate change and asthma, revealing thematic evolution from allergen and epidemiological studies toward mechanistic and policy‐oriented research. Strengthening interdisciplinary collaboration, enhancing global data sharing, and embedding asthma prevention into broader climate mitigation and adaptation frameworks will be critical for reducing the respiratory health burden in a changing climate.

## 1. Introduction

Climate change, characterized by rising global temperatures, increased frequency of extreme weather events, and altered environmental conditions, has emerged as one of the most pressing public health challenges of the 21st century [1, 2]. Beyond its well‐documented impacts on ecosystems, agriculture, and infrastructure, climate change poses substantial threats to human health, particularly in the realm of respiratory diseases [3–5]. Asthma, a chronic inflammatory disorder of the airways affecting more than 260 million people worldwide, is highly sensitive to environmental triggers, making it particularly vulnerable to the direct and indirect effects of climate change [3, 6].

Multiple pathways link climate change to asthma exacerbations and morbidity. These include increased exposure to aeroallergens such as pollen and mold spores, elevated levels of air pollutants including particulate matter (PM2.5) and ozone, and greater variability in temperature and humidity [7–9]. Extreme weather events, such as heatwaves, wildfires, and thunderstorms, have been associated with surges in asthma‐related emergency visits and hospitalizations [10, 11]. Moreover, changes in atmospheric conditions can alter allergen production, distribution, and potency, further compounding the risk for individuals with asthma [5, 12–14].

In recent years, a growing body of research has explored these complex interactions, spanning disciplines such as environmental science, epidemiology, immunology, and public health policy. However, despite this rapid expansion of literature, the field remains fragmented, with studies dispersed across various topics, regions, and methodologies. Traditional narrative reviews, while valuable, often lack the capacity to capture the evolving structure, emerging trends, and interdisciplinary nature of this research area.

Bibliometric analysis, which combines quantitative evaluation with visual mapping techniques, offers a powerful approach to systematically analyze the development, hotspots, and frontiers of scientific research [15]. This method enables the identification of influential publications, leading authors, collaborative networks, and thematic evolution over time, thereby providing a comprehensive understanding of the intellectual structure of a given field. When applied to climate change and asthma research, bibliometric analysis can reveal interdisciplinary linkages, highlight emerging areas of interest, and support evidence‐based decision‐making for both research prioritization and policy formulation.

In parallel with the growing scientific interest, global policy frameworks have increasingly recognized the health implications of climate change. The Paris Agreement (2015) underscored the urgency of limiting global warming to well below 2°C, with co‐benefits for public health through improved air quality and reduced environmental exposures [16]. The World Health Organization (WHO) has emphasized the integration of climate change mitigation and adaptation strategies into national health agendas, highlighting respiratory diseases, including asthma, as priority conditions [17, 18]. Initiatives such as the WHO Global Platform on Climate Change and Health and regional climate–health action plans have encouraged interdisciplinary collaboration, evidence‐based policymaking, and the development of early warning systems for climate‐sensitive diseases. However, translating these global commitments into targeted interventions for asthma remains a challenge, partly due to the fragmented nature of the existing evidence base and the need for cross‐sectoral coordination.

Therefore, this study aimed to perform a comprehensive bibliometric and visualized analysis of the global research landscape on climate change and asthma over the past two decades. Using the Web of Science Core Collection (WoSCC) as the primary data source and CiteSpace for visualization, we mapped the collaborative networks, identified core contributors, and explored the evolution of research hotspots and emerging frontiers. The findings of this study are expected to guide researchers, clinicians, and policymakers in advancing the understanding of climate change–related asthma and in developing effective prevention and adaptation strategies.

## 2. Materials and Methods

### 2.1. Data Sources and Search Strategy

The literature dataset for this bibliometric analysis was retrieved from the WoSCC, which is recognized as one of the most authoritative and comprehensive databases for bibliometric studies. A topic search was conducted using the following search query: TS = (“climate change” OR “global warming” OR “extreme heat” OR heatwave OR “ambient temperature” OR “temperature variability” OR “extreme weather” OR “thermal stress” OR “environmental temperature”) AND TS = (asthma OR “bronchial asthma” OR “childhood asthma” OR “pediatric asthma”).

**FIGURE 1:**
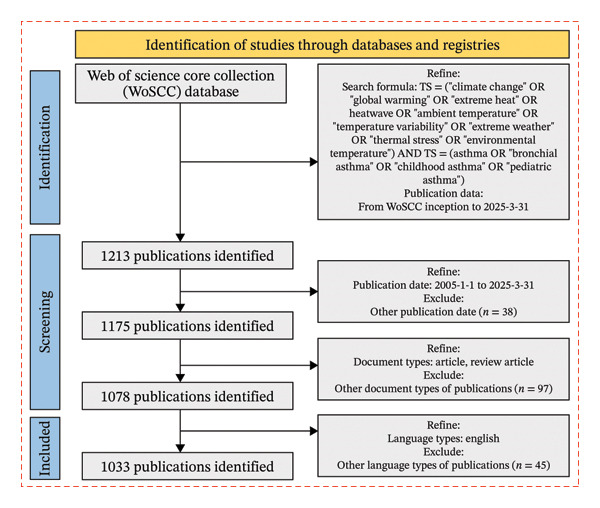
Flow diagram of the literature screening process.

The initial search returned 1213 records without any restrictions on language, publication type, or timespan. To focus on the most relevant and high‐quality research, the dataset was refined by limiting the timespan to publications from January 1, 2005, to March 31, 2025, which yielded 1175 records; restricting the document type to articles and reviews, resulting in 1078 records; and selecting only English‐language publications, producing the final dataset of 1033 records. All bibliographic information, including authors, titles, abstracts, keywords, institutions, countries/regions, references, and cited references, was downloaded in plain text format with full records and cited references for subsequent analysis. The literature screening process is illustrated in Figure 1.

### 2.2. Data Analysis and Visualization

All bibliometric analyses and visualizations were performed using CiteSpace (Version 6.1.R6), a software widely applied for detecting trends and patterns in scientific literature [19, 20]. The bibliographic data exported from WoSCC were imported into CiteSpace in plain text format with full records and cited references. The analysis covered the period from January 2005 to March 2025, with a time slice set to one year. The term source was set to include titles, abstracts, author keywords, and Keywords Plus.

The analysis focused on coauthorship (authors, institutions, and countries/regions), cocitation (references and journals), and co‐occurrence (keywords) to reveal the collaborative networks, intellectual structure, and research hotspots of the field. Node types were selected according to the specific analysis purpose, and the top 50 most‐cited or most‐occurring items from each slice were extracted. The pruning algorithms of “pathfinder” and “pruning sliced networks” were applied to simplify the networks and highlight the most significant connections. Key bibliometric indicators, including betweenness centrality, citation burst detection, and clustering evaluation metrics, were used to assess the structure and interpretability of the networks. Betweenness centrality was used to identify pivotal nodes linking different parts of the network, whereas citation burst detection was applied to detect references or keywords that received rapidly increased attention over a specific period. Cluster consistency and homogeneity were evaluated using silhouette values. In general, a silhouette value greater than 0.5 indicates acceptable consistency, whereas a value greater than 0.7 suggests high reliability of the clustering structure.

## 3. Results

### 3.1. Annual Publication Trends

**FIGURE 2:**
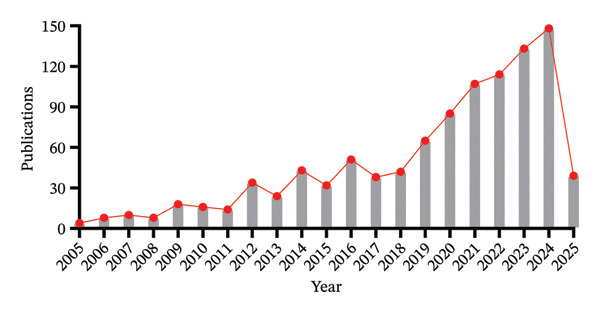
Annual publication trends in research on climate change and asthma from 2005 to 2025.

From 2005 to 2025, the number of publications related to climate change and asthma demonstrated a generally increasing trend, with notable fluctuations in certain years (Figure 2). Between 2005 and 2011, the annual output remained relatively low, with fewer than 20 publications per year. A marked increase began in 2012, followed by intermittent fluctuations until 2018. Since 2019, the field has experienced a sustained and substantial growth, reaching its peak in 2024 with 148 publications. Although the number of publications in 2025 (as of 31 March) appears lower than the previous year, this is likely due to incomplete data collection for the current year. Overall, the upward trajectory indicates growing scholarly attention and research activity on the intersection of climate change and asthma over the past two decades.

### 3.2. Countries/Regions and Institutions Analysis

**FIGURE 3:**
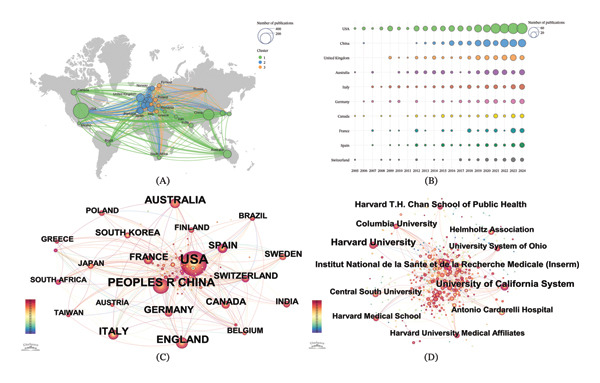
Analysis of countries/regions and institutions involved in climate change and asthma research. (A) Global collaboration map of countries/regions, with node size representing the number of publications and lines indicating collaborative links. (B) Annual publication trends of the top 10 most productive countries from 2005 to 2024. (C) Country co‐occurrence network showing collaboration patterns and publication volume. (D) Institutional co‐occurrence network, with larger nodes indicating higher publication output and more central positions representing greater influence in the research network.

The geographical distribution and collaboration network of research on climate change and asthma are shown in Figure 3 and Table S1. The country collaboration map (Figure 3A) revealed extensive international cooperation, with particularly strong collaborative links between the United States, China, Australia, the United Kingdom, and Spain. The temporal distribution of publications in the top 10 most productive countries (Figure 3B) indicated that the United States consistently led in annual outputs, followed by China and the United Kingdom, with marked growth in publication counts after 2015 in most countries. The country co‐occurrence network (Figure 3C) further confirmed the central role of the United States and China in this research field, both in terms of publication volume and international collaboration. Other influential contributors included England, Australia, Germany, Italy, and Canada, all of which demonstrated close links with multiple countries.

Institutional collaboration analysis (Figure 3D) showed that the most productive and influential institutions included Harvard T.H. Chan School of Public Health, Harvard University, Columbia University, the University of California System, and the Helmholtz Association. The presence of diverse institutions from North America, Europe, Asia, and Oceania reflects the multidisciplinary and global nature of research on climate change and asthma.

### 3.3. Author Analysis

**FIGURE 4:**
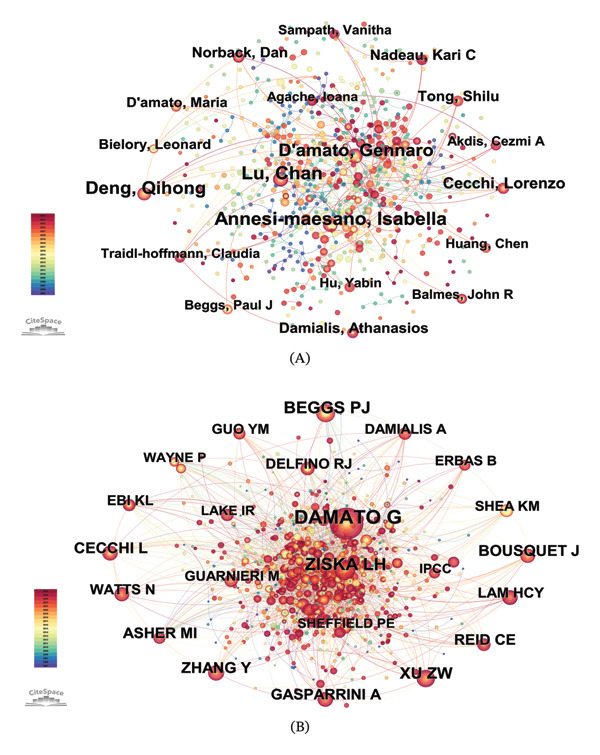
Author analysis in climate change and asthma research. (A) Author coauthorship network, where node size represents the number of publications and lines indicate collaborative relationships among authors. (B) Author co‐citation network, with larger nodes indicating higher cocitation frequency and more central positions representing greater influence in the knowledge structure of the field.

The coauthorship network (Figure 4A and Table S2) revealed that research on climate change and asthma involves a broad and diverse group of scholars from multiple disciplines and regions. Among them, D’Amato Gennaro, Lu Chan, and Annesi‐Maesano Isabella emerged as the most prolific authors, each exhibiting extensive collaborative networks. Other notable contributors included Deng Qihong, Beggs Paul J, Damialis Athanasios, and Traidl‐Hoffmann Claudia, who also demonstrated strong connections with international research teams. The dense interlinkages among nodes indicate a relatively high degree of collaboration within the field, although some regional clusters remain evident.

The cocited author network (Figure 4B and Table S2) highlighted D’Amato Gennaro as the most frequently cited scholar, underscoring his significant influence on the intellectual structure of the field. Other highly co‐cited authors included Ziska Lewis H, Beggs Paul J, Gasparrini Antonio, Reid Colleen E, and Sheffield Perry E, reflecting a concentration of key contributions from experts in environmental epidemiology, climate science, and respiratory health. The prominent position of authors from both clinical and environmental research backgrounds suggests that the field of climate change and asthma is characterized by strong interdisciplinary integration.

### 3.4. Journal Analysis

**FIGURE 5:**
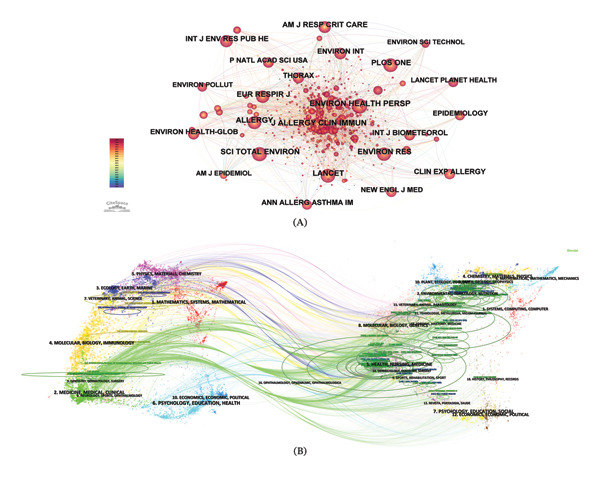
Journal analysis in climate change and asthma research. (A) Journal cocitation network, where node size indicates the cocitation frequency of a journal and connections represent cocitation relationships. (B) Dual‐map overlay of journals, illustrating citation trajectories between disciplines. The left side represents the citing journals, and the right side represents the cited journals.

The journal cocitation network (Figure 5A and Table S3) demonstrated that research on climate change and asthma is highly interdisciplinary, with influential journals spanning environmental sciences, respiratory medicine, epidemiology, and public health. Environmental Health Perspectives, Science of the Total Environment, and Environment International were the most frequently cocited journals, indicating their central role in disseminating research findings in this field. Other prominent journals included The Lancet, European Respiratory Journal, Allergy, and American Journal of Respiratory and Critical Care Medicine, reflecting the combined influence of environmental and clinical research sources. The close interconnections among journals suggest a high degree of cross‐disciplinary citation and collaboration.

The dual‐map overlay of journals (Figure 5B) revealed the citation trajectories between disciplines. Articles published in journals located in the fields of medicine, medical, clinical and health, nursing were most frequently cited by journals in environmental, toxicology, nutrition and earth and environmental, multidisciplinary domains. This pattern illustrates the multidisciplinary nature of climate change and asthma research, where environmental science serves as a critical foundation for clinical and public health applications. The visualization also highlighted emerging linkages between environmental health journals and those in molecular biology, immunology, and genetics, suggesting an increasing integration of mechanistic studies into population‐level research.

### 3.5. Reference Cocitation Analysis

**FIGURE 6:**
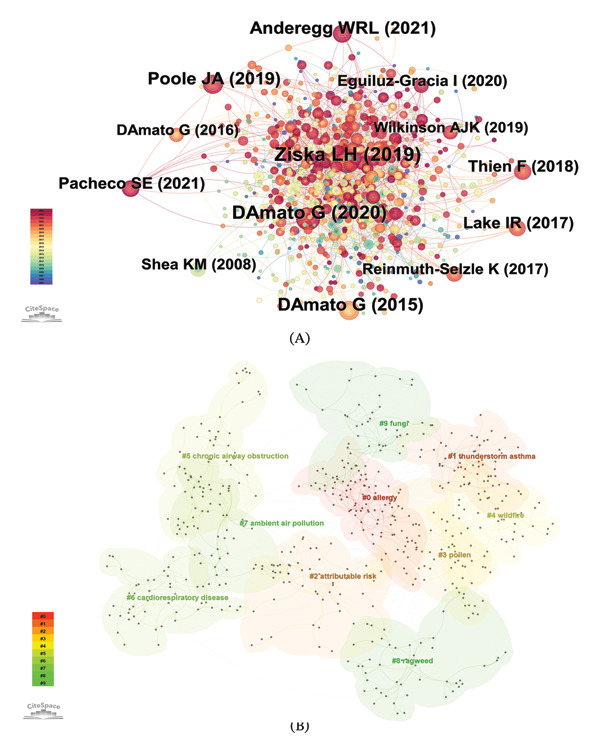
Reference cocitation analysis in climate change and asthma research. (A) Reference cocitation network, where node size represents the frequency of co‐citation and connections indicate cocitation relationships. (B) Cluster analysis of cocited references, showing major thematic clusters.

The reference cocitation network (Figure 6A and Table S4) revealed that several key publications have played a pivotal role in shaping the research landscape on climate change and asthma. Among them, works by D’Amato G (2015, 2016, 2020), Ziska LH (2019), and Beggs PJ (2004, 2005, 2010) were highly co‐cited, indicating their foundational influence on the field. Other frequently co‐cited references included studies by Shea KM (2008), Lake IR (2017), and Thien F (2018), which collectively covered a broad range of topics from climate‐related environmental exposures to respiratory health outcomes. Cluster analysis of co‐cited references (Figure 6B) identified several major thematic areas, such as thunderstorm asthma (#1), pollen (#3), wildfire (#4), chronic airway obstruction (#5), ambient air pollution (#7), ragweed (#8), and fungi (#9). These clusters reflect the diverse environmental triggers and pathophysiological mechanisms linking climate change to asthma exacerbations. The presence of clusters related to attributable risk (#2) and cardiorespiratory disease (#6) highlights the growing integration of epidemiological risk assessment into this research domain. Detailed information on the reference cocitation clusters is provided in Table S5.

**FIGURE 7:**
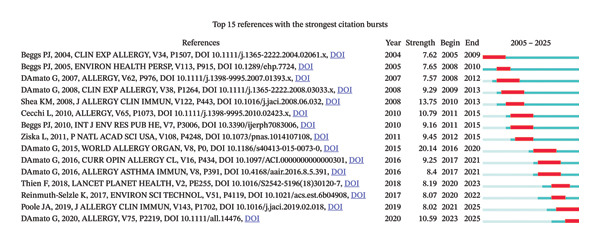
Top 15 references with the strongest citation bursts in climate change and asthma research from 2005 to 2025.

The citation burst analysis (Figure 7) further pinpointed references that have attracted exceptional attention within specific timeframes. The strongest burst was observed for D’Amato G (2015) in the World Allergy Organization Journal (strength = 20.14, 2016–2020), followed by influential works such as Shea KM (2008), Cecchi L (2010), and Ziska L (2011). More recent bursts include Poole JA (2019), D’Amato G (2020), and Reinmuth‐Selzle K (2017), indicating an ongoing research emphasis on climate‐sensitive allergens, extreme weather events, and mechanistic insights into asthma pathogenesis. The temporal patterns of these bursts suggest a shift from early conceptual frameworks toward applied and mechanistic research in recent years.

The temporal evolution of highly cited and burst references indicates that research on climate change and asthma has undergone a clear thematic transition over the past two decades. Early studies (2004–2010) primarily established conceptual links between environmental factors such as aeroallergens, temperature variability, and air pollution and asthma morbidity, laying the groundwork for the field. From 2011 to 2017, the focus expanded to include epidemiological evidence from extreme weather events, such as heatwaves and thunderstorm asthma outbreaks, as well as the role of specific allergenic sources like pollen and fungal spores. More recently (2018–2025), there has been a marked shift toward mechanistic and interdisciplinary investigations, integrating environmental science with immunology, molecular biology, and predictive modeling to elucidate biological pathways and quantify the health burden attributable to climate change. This evolution reflects a growing emphasis on actionable research that informs public health interventions, early warning systems, and climate adaptation policies.

### 3.6. Keyword Analysis

**FIGURE 8:**
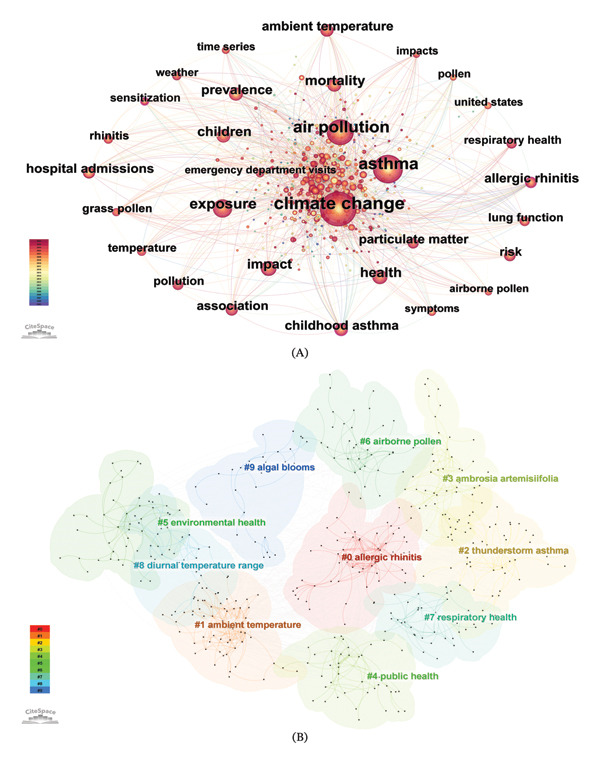
Keyword analysis in climate change and asthma research. (A) Keyword co‐occurrence network, where node size represents keyword frequency and connections indicate co‐occurrence relationships. (B) Keyword clustering analysis.

The keyword co‐occurrence network (Figure 8A) identified “climate change,” “asthma,” “air pollution,” “ambient temperature,” “allergic rhinitis,” and “childhood asthma” as the most frequently occurring terms, indicating the central research themes linking environmental factors to respiratory health outcomes. Other high‐frequency keywords, such as particulate matter, pollen, respiratory health, and hospital admissions, reflect the multifaceted pathways through which climate change influences asthma morbidity. Keyword clustering analysis (Figure 8B) revealed 10 major thematic clusters, including #0 allergic rhinitis, #1 ambient temperature, #2 thunderstorm asthma, #3 *Ambrosia artemisiifolia*, #4 public health, #5 environmental health, #6 airborne pollen, #7 respiratory health, #8 diurnal temperature range, and #9 algal blooms. These clusters encompass both direct triggers of asthma exacerbations (e.g., aeroallergens, temperature fluctuations, extreme weather events) and broader public health concerns (e.g., environmental health, community‐level risk management). Detailed information on the keyword clusters is provided in Table S6.

**FIGURE 9:**
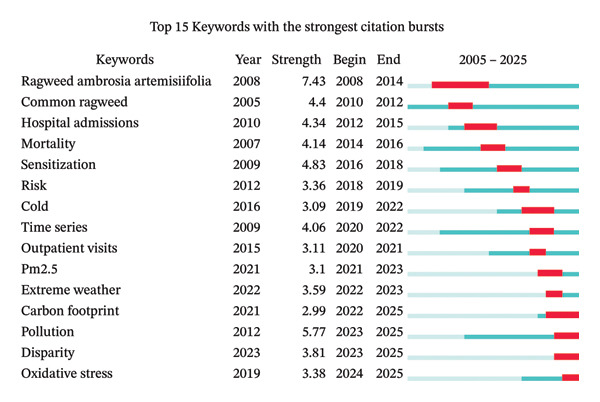
Top 15 keywords with the strongest citation bursts in climate change and asthma research from 2005 to 2025.

The top 15 keywords with the strongest citation bursts (Figure 9) demonstrated a temporal shift in research priorities. Early bursts focused on allergenic triggers such as “ragweed *Ambrosia artemisiifolia*” (2008–2014) and “common ragweed” (2010–2012). Subsequent bursts reflected increasing attention to health outcomes and risk factors, including “hospital admissions” (2012–2015), “mortality” (2014–2016), and “sensitization” (2016–2018). More recent bursts have centered on environmental exposures and emerging health threats, such as “pm2.5” (2021–2023), “extreme weather” (2022–2023), “carbon footprint” (2022–2025), “pollution” (2023–2025), and “oxidative stress” (2024–2025), indicating a growing mechanistic and preventive research focus.

**FIGURE 10:**
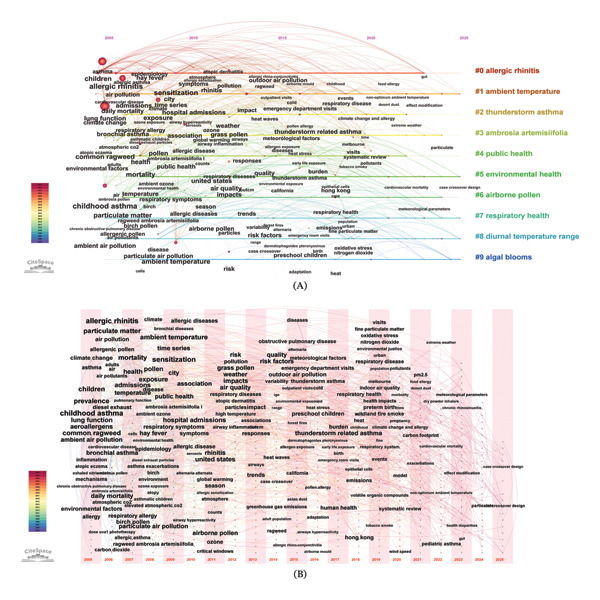
Temporal evolution of research hotspots in climate change and asthma. (A) Keyword timeline view, where clusters are arranged along the vertical axis and time is represented on the horizontal axis. The size of the nodes reflects keyword frequency, and connections indicate temporal co‐occurrence patterns. (B) Keyword timezone view, illustrating the chronological appearance and persistence of keywords from 2005 to 2025.

The keyword timeline view (Figure 10A) illustrated the chronological development of research hotspots. Early research (2005–2012) concentrated on allergic diseases and environmental triggers, while the middle phase (2013–2018) expanded to encompass air quality, meteorological variables, and public health outcomes. The most recent phase (2019–2025) has increasingly integrated molecular and mechanistic terms (e.g., oxidative stress) alongside broader policy‐oriented topics (e.g., carbon footprint, climate adaptation). The keyword timezone view (Figure 10B) further emphasized the progressive evolution from allergen‐specific studies toward interdisciplinary approaches combining environmental science, epidemiology, and molecular biology. This evolution reflects a shift in the field’s research agenda from documenting associations to elucidating biological mechanisms and informing evidence‐based policy and intervention strategies.

Overall, the keyword analysis reveals a dynamic evolution of research on climate change and asthma over the past two decades. The focus has shifted from early observational studies on allergen exposure and respiratory outcomes to more complex, interdisciplinary investigations integrating environmental monitoring, epidemiological modeling, and molecular biology. Emerging hotspots such as extreme weather, PM2.5, carbon footprint, pollution, and oxidative stress highlight a growing interest in understanding the mechanistic pathways linking climate‐related environmental changes to asthma exacerbations. Simultaneously, the appearance of terms related to public health and climate adaptation reflects an increasing emphasis on translating scientific evidence into policy‐making and preventive interventions. These trends suggest that future research is likely to prioritize predictive modeling of climate‐health interactions, elucidation of biological mechanisms, and evaluation of intervention strategies to mitigate the respiratory health impacts of climate change.

## 4. Discussion

### 4.1. Overview of Major Findings

This bibliometric and visualized analysis comprehensively mapped the global research landscape on climate change and asthma from 2005 to 2025. The number of publications has grown steadily, with a marked increase after 2015 and a peak in 2024, reflecting the rising recognition of climate change as a critical determinant of respiratory health. The United States and China were the most prolific contributors, both in terms of publication output and collaborative networks, followed by the United Kingdom, Australia, and Germany. Institutional analysis revealed that leading contributors such as Harvard T.H. Chan School of Public Health, Harvard University, and Columbia University have maintained extensive global collaborations, while several Asian institutions, including Central South University, have emerged as important players in recent years. Author analysis identified D’Amato Gennaro, Lu Chan, and Annesi‐Maesano Isabella as the most productive authors, with D’Amato Gennaro, Ziska Lewis H, and Beggs Paul J being the most frequently co‐cited.

Journal cocitation analysis showed that Environmental Health Perspectives, Science of the Total Environment, and Environment International are the most influential journals in this field. Reference cocitation and cluster analyses revealed thematic foci on thunderstorm asthma, pollen, wildfire smoke, and ambient air pollution, with highly cited works by D’Amato G (2015, 2016, 2020) and Ziska LH (2019) serving as pivotal contributions. Keyword analyses indicated a transition in research priorities from allergen‐related and epidemiological studies to interdisciplinary, mechanistic, and policy‐oriented research, highlighting the dynamic evolution of this domain.

### 4.2. Research Hotspots

The results demonstrate that research on climate change and asthma has concentrated on several interrelated thematic areas. First, climate‐related environmental exposures remain a dominant focus. Air pollutants such as PM2.5, ozone, and nitrogen dioxide have been consistently associated with asthma exacerbations, while meteorological factors including ambient temperature, humidity, and diurnal temperature range have been shown to influence airway inflammation and disease severity [21, 22]. Extreme weather events, notably heatwaves, wildfires, and thunderstorms, have been implicated in sudden surges of asthma‐related emergency visits, with “thunderstorm asthma” identified as a major cluster (#2) in keyword analysis.

Second, allergen dynamics and immunological mechanisms have gained increasing attention. Climatic changes influence pollen production, allergenicity, and seasonal patterns, while higher CO_2_ concentrations and temperatures may enhance allergen potency [23–25]. Ragweed (*Ambrosia artemisiifolia*) and fungal spores, highlighted in clusters #3 and #9, exemplify the role of specific biological agents in asthma pathophysiology [26, 27]. Mechanistic research has increasingly examined oxidative stress, epithelial barrier dysfunction, and immune modulation as mediators between climate change and asthma morbidity [28–30]. Third, vulnerable populations, particularly children, are a recurrent research priority. The term “childhood asthma” appears frequently in co‐occurrence maps, and pediatric populations are disproportionately affected by climate‐sensitive triggers due to developing airways and immune systems.

Finally, public health and epidemiological evidence linking climate change to asthma burden has expanded, with growing interest in quantifying attributable risks, forecasting future health impacts, and integrating asthma into climate adaptation strategies.

In addition, the research landscape appears to show some degree of geographic heterogeneity. Although climate change is a global phenomenon, its environmental manifestations and health impacts differ across regions, which likely shapes local research priorities. For example, wildfire‐related asthma studies appear to be particularly prominent in North America and Australia, whereas pollen‐ and ragweed‐related research has been especially visible in Europe and North America. In contrast, studies focusing on air pollution, PM2.5, ozone, and urban environmental exposures have been more frequently represented in East Asia. These regional patterns likely reflect differences in climate‐sensitive exposures, ecological conditions, urbanization, and public health concerns. However, more explicit region‐topic coupling analyses are still needed to better characterize the geographic distribution of specific research frontiers.

### 4.3. Emerging Research Frontiers

This study identified several research frontiers shaping the future of the field. One major trend is interdisciplinary integration, bringing together environmental science, epidemiology, molecular biology, and public health policy to address the complex pathways linking climate change and asthma. Cocitation and keyword timezone analyses indicate that the field is moving beyond descriptive associations toward mechanistic and translational research. Predictive modeling and early warning systems represent another frontier. By integrating meteorological forecasts, air quality monitoring, and health surveillance data, predictive models could identify high‐risk periods for asthma exacerbations and enable proactive interventions. Climate adaptation and health interventions are also gaining momentum. Global frameworks such as the WHO’s Global Platform on Climate Change and Health emphasize the need for targeted strategies to reduce exposure, strengthen healthcare systems, and increase community resilience. In the context of asthma, such measures may include pollen and air quality alerts, public education campaigns, and urban planning initiatives to mitigate environmental triggers.

At the molecular level, oxidative stress and immune regulation have emerged as key mechanistic themes, reflecting a shift toward understanding the biological processes that mediate environmental effects on asthma [31, 32]. Integrating genomics, proteomics, and metabolomics with environmental exposure data could yield novel biomarkers and therapeutic targets [33, 34]. Finally, sustainable development and mitigation strategies such as reducing carbon footprints, promoting green infrastructure, and transitioning to clean energy hold promise for simultaneously addressing climate change and reducing asthma burden.

### 4.4. Challenges and Future Directions

Despite significant progress, several challenges remain in advancing research on climate change and asthma. One major limitation is the fragmentation of evidence, as most studies are region‐specific or short‐term in nature, with few integrating data from multiple continents. This restricts the ability to synthesize global patterns and draw generalizable conclusions. Establishing multinational, long‐term cohorts with harmonized methodologies would improve comparability and provide more robust evidence for understanding the global burden of asthma in the context of climate change. In addition, mechanistic research remains underdeveloped compared with the extensive body of epidemiological studies. There is a pressing need for experimental and clinical research to validate causal pathways, elucidate biological mechanisms such as oxidative stress and immune modulation, and identify modifiable risk factors that could be targeted for prevention or intervention.

Another critical challenge lies in policy implementation and cross‐sectoral collaboration. Although climate–health frameworks have been established at global and national levels, asthma‐specific adaptation measures are rarely integrated into climate action plans. This gap limits the translation of scientific evidence into effective public health interventions. Future research should aim to strengthen interdisciplinary and international collaborations, develop and validate climate–health prediction and early warning systems, and conduct intervention trials in high‐risk populations to assess the effectiveness of preventive measures. Embedding asthma management into broader climate adaptation and mitigation strategies will be essential for reducing disease burden and improving resilience to the health impacts of climate change.

### 4.5. Strengths and Limitations

This study has several strengths. It is, to our knowledge, the first to systematically map the global research landscape on climate change and asthma over a 20‐year period using bibliometric and visualization techniques. The approach allowed us to identify key contributors, collaborative networks, thematic evolution, and emerging frontiers in an objective and reproducible manner.

However, certain limitations should be acknowledged. First, the analysis was based solely on the WOSCC, which may have excluded relevant studies indexed in other databases. Second, bibliometric methods cannot substitute for systematic reviews or meta‐analyses in evaluating study quality or effect sizes. Third, the 2025 data are incomplete, potentially underestimating publication counts and recent trends.

### 4.6. Future Research Priorities

Although the current literature has substantially advanced understanding of the links between climate change and asthma, several key questions remain insufficiently addressed. First, future studies should move beyond region‐specific and short‐term analyses toward multinational, longitudinal, and standardized cohort designs to improve comparability across different climatic, environmental, and socioeconomic settings. Second, more work is needed to disentangle the independent and interactive effects of climate‐related factors, including air pollution, aeroallergens, temperature variability, and extreme weather events, on asthma incidence, exacerbations, and disease severity. Third, vulnerable populations, particularly children, older adults, and socioeconomically disadvantaged communities, require more focused investigation to better define susceptibility profiles and inequities in climate‐related asthma burden. Fourth, the field would benefit from stronger integration of epidemiological evidence with mechanistic studies, including oxidative stress, epithelial injury, immune dysregulation, and multiomics approaches, to identify biologically meaningful pathways and potential biomarkers. Fifth, future research should prioritize the development and validation of predictive models and early warning systems that combine environmental monitoring, meteorological forecasting, and health data to support timely preventive interventions. Finally, greater attention should be given to implementation and policy translation, including evaluation of adaptation strategies, public health messaging, and healthcare system responses that can reduce asthma burden under changing climate conditions.

## 5. Conclusions

This bibliometric and visualized analysis mapped the global research landscape on climate change and asthma over the past two decades, revealing a steady increase in publications, especially after 2015, and highlighting the United States and China as the most prolific contributors. Thematic analyses identified key research hotspots, including climate‐related environmental exposures, allergen dynamics, vulnerable populations, and public health impacts, as well as emerging frontiers such as predictive modeling, mechanistic studies, and climate adaptation strategies. Despite substantial progress, challenges remain in addressing the fragmentation of evidence, advancing mechanistic research, and integrating asthma‐specific measures into climate–health policies. Strengthening interdisciplinary collaboration, enhancing global data sharing, and embedding asthma prevention into broader climate mitigation and adaptation efforts will be critical for reducing the respiratory health burden in a changing climate.

## Author Contributions

Yixin Wang, Feng Jiang, and Jin Shu conceived and designed the study. Yixin Wang, Qi Liu, Jiaxin Cao, and Yuxin Liu collected and analyzed the data. Huanxia Qu contributed to data interpretation and visualization. Xinchen Shu contributed to data cleaning, software‐based bibliometric analysis, statistical/network analysis, and interpretation of quantitative results. Yixin Wang drafted the manuscript. Feng Jiang and Jin Shu critically revised the manuscript and supervised the study.

## Funding

The authors declare that no funds, grants, or other support were received during the preparation of this manuscript.

## Disclosure

All authors read and approved the final version of the manuscript.

## Ethics Statement

This study is a bibliometric analysis based on previously published literature and does not involve human participants or animals.

## Consent

The authors have nothing to report.

## Conflicts of Interest

The authors declare no conflicts of interest.

## Supporting Information

Additional supporting information can be found online in the Supporting Information section.

## Supporting information


**Supporting Information 1** Table S1. Top 10 countries and institutions contributing to climate change and asthma research.

**Supporting Information 2** Table S2. Top 10 authors and cocited authors in climate change and asthma research.

**Supporting Information 3** Table S3. Top 10 journals and cocited journals in climate change and asthma research.

**Supporting Information 4** Table S4. Top 10 highly cocited references in climate change and asthma research.

**Supporting Information 5** Table S5. Summary of reference cocitation clusters in climate change and asthma research.

**Supporting Information 6** Table S6. Summary of keyword clusters in climate change and asthma research.

## Data Availability

The datasets generated and/or analyzed during the current study are available from the corresponding author on reasonable request.
