# Supplementary material for: Emerging Trends and Research Frontiers in Climate Change and Asthma: Insights From a Two‐Decade Bibliometric Analysis
Source: Can Respir J. 2026 Jun 22;2026:5546333. doi: 10.1155/carj/5546333 (PMC13287831; doi:10.1155/carj/5546333)
Supplement: Supplementary file 1 — Supporting Information 1 Table S1. Top 10 countries and institutions contributing to climate change and asthma research. [file CARJ-2026-5546333-s001.docx]

**Table S1**. Top 10 countries and institutions contributing to climate change and asthma research.

| Rank | Country | Count | Institution | Count |
| --- | --- | --- | --- | --- |
| 1 | United States | 398 (38.5%) | Columbia University (United States) | 29 (2.8%) |
| 2 | China | 169 (16.3%) | Central South University (China) | 24 (2.3%) |
| 3 | Australia | 100 (9.6%) | Queensland University of Technology (Australia) | 23 (2.2%) |
| 4 | United Kingdom | 98 (9.4%) | Fudan University (China) | 22 (2.1%) |
| 5 | Italy | 83 (8.0%) | Anhui Medical University (China) | 21 (2.1%) |
| 6 | Germany | 67 (6.4%) | Boston University (United States) | 20 (1.9%) |
| 7 | Spain | 58 (5.6%) | Uppsala University (Sweden) | 19 (1.8%) |
| 8 | France | 57 (5.5%) | Tsinghua University (China) | 19 (1.8%) |
| 9 | Canada | 57 (5.5%) | Harvard Medical School (United States) | 19 (1.8%) |
| 10 | Switzerland | 44 (4.2%) | Harvard University (United States) | 19 (1.8%) |
