# Supplementary material for: Emerging Trends and Research Frontiers in Climate Change and Asthma: Insights From a Two‐Decade Bibliometric Analysis
Source: Can Respir J. 2026 Jun 22;2026:5546333. doi: 10.1155/carj/5546333 (PMC13287831; doi:10.1155/carj/5546333)
Supplement: Supplementary file 2 — Supporting Information 2 Table S2. Top 10 authors and cocited authors in climate change and asthma research. [file CARJ-2026-5546333-s006.docx]

**Table S2**. Top 10 authors and co-cited authors in climate change and asthma research.

| Rank | Authors | Count | Co-cited authors | Citations |
| --- | --- | --- | --- | --- |
| 1 | Chan Lu | 21 | Gennaro D’amato | 841 |
| 2 | Qihong Deng | 21 | Lewis H Ziska | 294 |
| 3 | Gennaro D’amato | 17 | Paul J Beggs | 218 |
| 4 | Dan Norback | 14 | Zhiwei Xu | 203 |
| 5 | Isabella Annesi-Maesano | 14 | J Bousquet | 199 |
| 6 | Shilu Tong | 13 | Antonio Gasparrini | 158 |
| 7 | Zhuohui Zhao | 12 | Colleen E Reid | 126 |
| 8 | Chen Huang | 12 | Lorenzo Cecchi | 120 |
| 9 | Xin Zhang | 12 | Yue Zhang | 116 |
| 10 | Maria D’amato | 11 | M I Asher | 99 |
