# Supplementary material for: Emerging Trends and Research Frontiers in Climate Change and Asthma: Insights From a Two‐Decade Bibliometric Analysis
Source: Can Respir J. 2026 Jun 22;2026:5546333. doi: 10.1155/carj/5546333 (PMC13287831; doi:10.1155/carj/5546333)
Supplement: Supplementary file 3 — Supporting Information 3 Table S3. Top 10 journals and cocited journals in climate change and asthma research. [file CARJ-2026-5546333-s005.docx]

**Table S3**. Top 10 journals and co-cited journals in climate change and asthma research.

| Rank | Journal | Count | IF | JCR | Co-cited Journal | Co-citation | IF | JCR |
| --- | --- | --- | --- | --- | --- | --- | --- | --- |
| 1 | Science of the Total Environment | 49 (4.7%) | 8.0 | Q1 | Journal of Allergy and Clinical Immunology | 2681 | 11.2 | Q1 |
| 2 | Environmental Research | 45 (4.3%) | 7.7 | Q1 | Allergy | 1966 | 12.0 | Q1 |
| 3 | International Journal of Environmental Research and Public Health | 31 (3.0%) | / | / | Environmental Health Perspectives | 1845 | 9.8 | Q1 |
| 4 | International Journal of Biometeorology | 22 (2.1%) | 2.6 | Q2 | Science of the Total Environment | 1285 | 8.0 | Q1 |
| 5 | Journal of Allergy and Clinical Immunology | 21 (2.0%) | 11.2 | Q1 | Environmental Research | 1271 | 7.7 | Q1 |
| 6 | Environment International | 20 (1.9%) | 9.7 | Q1 | Lancet | 1135 | 88.5 | Q1 |
| 7 | Plos One | 20 (1.9%) | 2.6 | Q2 | Clinical and Experimental Allergy | 1069 | 5.2 | Q1 |
| 8 | Annals of Allergy asthma & Immunology | 18 (1.7%) | 4.7 | Q1 | American Journal of Respiratory and Critical Care Medicine | 991 | 19.4 | Q1 |
| 9 | Allergy | 16 (1.5%) | 12.0 | Q1 | European Respiratory Journal | 962 | 21.0 | Q1 |
| 10 | Environmental Health | 16 (1.5%) | 5.9 | Q1 | International Journal of Biometeorology | 891 | 2.6 | Q2 |
