# Supplementary material for: Emerging Trends and Research Frontiers in Climate Change and Asthma: Insights From a Two‐Decade Bibliometric Analysis
Source: Can Respir J. 2026 Jun 22;2026:5546333. doi: 10.1155/carj/5546333 (PMC13287831; doi:10.1155/carj/5546333)
Supplement: Supplementary file 4 — Supporting Information 4 Table S4. Top 10 highly cocited references in climate change and asthma research. [file CARJ-2026-5546333-s004.docx]

**Table S4**. Top 10 highly co-cited references in climate change and asthma research.

| Rank | Co-cited reference | Citations |
| --- | --- | --- |
| 1 | D'amato G, 2015, World Allergy Organ, V8 | 98 |
| 2 | Beggs PJ, 2004, Clin Exp Allergy, V34, P1507 | 79 |
| 3 | D'amato G, 2007, Allergy, V62, P976 | 79 |
| 4 | Ziska LH, 2003, J Allergy Clin Immun, V111, P290 | 76 |
| 5 | Shea KM, 2008, J Allergy Clin Immun, V122, P443 | 70 |
| 6 | Ziska LH, 2019, Lancet Planet Health, V3, Pe124 | 68 |
| 7 | Wayne P, 2002, Ann Allerg Asthma Im, V88, P279 | 67 |
| 8 | Cecchi L, 2010, Allergy, V65, P1073 | 62 |
| 9 | Ziska L, 2011, P Natl Acad Sci USA, V108, P4248 | 60 |
| 10 | Guarnieri M, 2014, Lancet, V383, P1581 | 60 |
