# Supplementary material for: Emerging Trends and Research Frontiers in Climate Change and Asthma: Insights From a Two‐Decade Bibliometric Analysis
Source: Can Respir J. 2026 Jun 22;2026:5546333. doi: 10.1155/carj/5546333 (PMC13287831; doi:10.1155/carj/5546333)
Supplement: Supplementary file 5 — Supporting Information 5 Table S5. Summary of reference cocitation clusters in climate change and asthma research. [file CARJ-2026-5546333-s003.docx]

**Table S5.** Summary of reference co-citation clusters in climate change and asthma research.

| **ClusterID** | **Size** | **Silhouette** | **mean(Year)** | **Label (LSI)** | **Label (LLR)** | **Label (MI)** |
| --- | --- | --- | --- | --- | --- | --- |
| 0 | 87 | 0.907 | 2020 | climate change; allergic diseases; air pollutants; airborne pollen; pollen allergy framework \| air pollution; global warming; vulnerable populations; dust storms; greenhouse gases | allergy (6.74, 0.01); nonallergic rhinitis (6.73, 0.01); climate change (6.63, 0.05); wildfire (4.06, 0.05); ozone (3.65, 0.1) | biologics (0.93); carbon di- oxide (0.93); direct effects (0.93); neophyte (0.93); ethnicity (0.93) |
| 1 | 56 | 0.872 | 2017 | climate change; allergic diseases; air pollutants; pollen allergy framework; airborne pollen \| air pollution; greenhouse gases; human health; dust storms; vulnerable populations | thunderstorm asthma (9, 0.005); asia-pacific (8.34, 0.005); epidemic thunderstorm asthma (8.34, 0.005); grass pollen (8.29, 0.005); aeroallergen (5, 0.05) | evaluation (0.57); pollen concentrations (0.57); national construction code (0.57); extreme pollen event (epe) (0.57); asthma treatment (0.57) |
| 2 | 56 | 0.962 | 2020 | climate change; extreme temperature; global warming; allergic diseases; epithelial barrier \| air pollution; covid-19 pandemic; pediatric asthma emergency room visits; time series analysis; airway inflammation | attributable risk (9.52, 0.005); effect modification (9.52, 0.005); preschool children (7.95, 0.005); allergy (6.87, 0.01); asthma (6.45, 0.05) | adolescent asthma (0.4); healthy behaviors (0.4); air temperature (0.4); montana (0.4); hospital discharge data (0.4) |
| 3 | 56 | 0.953 | 2008 | climate change; allergic diseases; thunderstorm-associated asthma; airway hyperreactivity; allergenic pollen \| air pollution; allergic sensitization; severe rhinitis; specific immunolglobulin; thunderstorm-associated asthma | pollen (7.22, 0.01); contact allergy (6.38, 0.05); severe rhinitis (6.38, 0.05); asthma bronchiale (6.38, 0.05); sinusitis (6.38, 0.05) | contact allergy (0.14); severe rhinitis (0.14); asthma bronchiale (0.14); sinusitis (0.14); food allergy (0.14) |
| 4 | 51 | 0.914 | 2019 | climate change; air pollution; dust storms; healthcare system; adaptive strategies \| respiratory health; greenhouse gases; urban heat island; lung neoplasms; healthcare system | wildfire (21.69, 1.0E-4); smoke (18.98, 1.0E-4); wildfires (12.16, 0.001); heat waves (7.88, 0.005); wildfire smoke (7.88, 0.005) | noncommunicable diseases (0.4); alaska (0.4); alaska native (0.4); extremely hot weather (0.4); interdisciplinary (0.4) |
| 5 | 51 | 0.966 | 2010 | case-crossover; temperature; season; asthma; respiratory symptoms \| outpatient visits; chronic airway obstruction; extreme temperature; respiratory symptoms; bayesian hierarchical model | chronic airway obstruction (9, 0.005); bayesian hierarchical model (9, 0.005); heat wave (9, 0.005); cold (9, 0.005); hospital admissions (9, 0.005) | asthma (0.04); climate change (0.04); chronic airway obstruction (0.03); bayesian hierarchical model (0.03); heat wave (0.03) |
| 6 | 50 | 0.868 | 2011 | climate change; allergic rhino-conjunctivitis; allergic conjunctivitis; vulnerable populations; allergic disease \| air pollution; airway hypersensitivity; pollen allergy; cardiorespiratory disease; pm2.5 | cardiorespiratory disease (6.77, 0.01); uncertainty quantification (6.77, 0.01); trans-disciplinary research (6.77, 0.01); exposure pathway (6.77, 0.01); forest fires (6.77, 0.01) | cardiorespiratory disease (0.11); uncertainty quantification (0.11); trans-disciplinary research (0.11); exposure pathway (0.11); forest fires (0.11) |
| 7 | 47 | 0.976 | 2015 | air pollution; preterm birth; childhood asthma; nitrogen dioxide; attention deficit hyperactivity disorder \| ambient air pollution; preschool children; atopic eczema; preterm birth; cohort study | ambient air pollution (19.7, 1.0E-4); pregnancy (18.83, 1.0E-4); china (15.27, 1.0E-4); cohort study (13.12, 0.001); trimester (13.1, 0.001) | preterm birth (ptb) (0.13); early-life (0.13); nitrogen dioxide (0.13); particulate matter (pm2.5) (0.13); trimesters (0.13) |
| 8 | 47 | 0.953 | 2005 | climate change; allergic rhinitis; mould spore; human health; allergic disease \| global warming; air pollution; mould spore; carbon dioxide; allergenic pollen | ragweed (10.27, 0.005); bronchial diseases (6.99, 0.01); climate variability (6.99, 0.01); cigarette smoke (6.99, 0.01); pediatric (6.99, 0.01) | bronchial diseases (0.1); climate variability (0.1); cigarette smoke (0.1); pediatric (0.1); springtime warming (0.1) |
| 9 | 40 | 0.959 | 2014 | climate change; viral respiratory tract infections; air pollutants; occupational asthma; optimal growing conditions \| air pollution; respiratory allergy; thunderstorm asthma; airway hyperreactivity; obstructive respiratory diseases | fungi (9.26, 0.005); climate change and respiratory allergy (7.56, 0.01); climate change and asthma (7.56, 0.01); experimental and observational models (6.46, 0.05); land uses (6.46, 0.05) | experimental and observational models (0.14); land uses (0.14); macvia-lr (0.14); information and communications technology (0.14); france (0.14) |

The table presents the main reference co-citation clusters identified by CiteSpace, including cluster size, silhouette value, mean publication year, and cluster label generated by the log-likelihood ratio (LLR) algorithm.
