# Supplementary material for: Emerging Trends and Research Frontiers in Climate Change and Asthma: Insights From a Two‐Decade Bibliometric Analysis
Source: Can Respir J. 2026 Jun 22;2026:5546333. doi: 10.1155/carj/5546333 (PMC13287831; doi:10.1155/carj/5546333)
Supplement: Supplementary file 6 — Supporting Information 6 Table S6. Summary of keyword clusters in climate change and asthma research. [file CARJ-2026-5546333-s002.docx]

**Table S6.** Summary of keyword clusters in climate change and asthma research.

| **ClusterID** | **Size** | **Silhouette** | **mean(Year)** | **Label (LSI)** | **Label (LLR)** | **Label (MI)** |
| --- | --- | --- | --- | --- | --- | --- |
| 0 | 65 | 0.805 | 2011 | climate change; air pollution; asthma; fungal spores; childhood \| allergic rhinitis; medical services; grass pollen; pm2.5; indoor air quality | allergic rhinitis (32.08, 1.0E-4); climate change (31.64, 1.0E-4); symptoms (22.79, 1.0E-4); allergy (21.96, 1.0E-4); prevalence (19.57, 1.0E-4) | carbon footprints (1.44); autonomic nervous system (1.44); anterior nares (1.44); amb a 6 (1.44); climate anxiety (1.44) |
| 1 | 64 | 0.737 | 2014 | air pollution; climate change; thunderstorm asthma; airway inflammation; pm2.5 \| ambient temperature; non-linear model; allergic rhinitis; transient receptor; potential ion channels | ambient temperature (28.42, 1.0E-4); morbidity (25.43, 1.0E-4); children (23.89, 1.0E-4); temperature (21.81, 1.0E-4); allergy (20.13, 1.0E-4) | healthy behaviors (0.78); effect modifier (0.78); hand (0.78); dlnm (0.78); exacerbation (0.78) |
| 2 | 52 | 0.839 | 2013 | climate change; allergic disease; food allergy; elevated atmospheric carbon dioxide; particle size \| respiratory allergy; allergic rhinitis; bronchial asthma; allergic asthma; allergenic pollens | thunderstorm asthma (46.36, 1.0E-4); respiratory allergy (39.01, 1.0E-4); pollen allergy (32.51, 1.0E-4); climate change and allergy (27.19, 1.0E-4); biodiversity and allergy (16.37, 1.0E-4) | allergenic pollens (0.31); ahvaz (0.31); fungal antigenic protein (0.31); climate change impact (0.31); interaction between climate change and allergy (0.31) |
| 3 | 49 | 0.812 | 2011 | climate change; allergic rhinitis; carbon dioxide; volatile organic carbons; water damage \| ambrosia artemisiifolia; allergenic pollen; vacant lots; spatial heterogeneity; environmental justice | ambrosia artemisiifolia (21.43, 1.0E-4); allergies (15.49, 1.0E-4); exhaled breath condensate (10.8, 0.005); mice (10.8, 0.005); ph (10.8, 0.005) | carbon di- oxide (0.32); sporulation (0.32); volatile organic carbons (0.32); invasion (0.32); enolase (0.32) |
| 4 | 42 | 0.799 | 2012 | air pollution; health impact assessment; traffic injuries; pm2.5; lung neoplasms \| climate change; ultrafine particles; oxidative stress; reactive oxygen species; diesel exhaust particles | public health (28.4, 1.0E-4); particulate matter (20.29, 1.0E-4); childhood asthma (6.05, 0.05); global warming (5.65, 0.05); cohort study (5.52, 0.05) | floods (0.6); texas (0.6); ambient temperature change (0.6); population health (0.6); fine particle (0.6) |
| 5 | 40 | 0.757 | 2016 | climate change; human health; air quality; skin cancer; food quality \| air pollution; monitoring compliance; personal air monitoring; exposure assessment; impacts | environmental health (13.54, 0.001); chronic disease (10.66, 0.005); emergency preparedness (10.66, 0.005); air quality (7.92, 0.005); environmental exposure (7.92, 0.005) | general practitioner (0.34); youth (0.34); wearable device (0.34); airborne dust (0.34); sustainable urbanization (0.34) |
| 6 | 37 | 0.827 | 2013 | climate change; airborne pollen; allergic diseases; air pollutants; pollen allergy framework \| air pollution; fungal sensitization; atmosphere; allergen; fine particulate matter | airborne pollen (27.9, 1.0E-4); pollen (13.32, 0.001); birch (11.21, 0.001); allergic diseases (7.28, 0.01); switzerland (6.67, 0.01) | long-distance transport (0.22); heat acclimation (0.22); earth system model (0.22); alpine environment (0.22); mediterranean area (0.22) |
| 7 | 36 | 0.81 | 2018 | climate change; health equity; public health; hazardous air pollutants; wildfire smoke \| air pollution; case-crossover study; ambient temperature; childhood asthma; early life | respiratory health (24.27, 1.0E-4); wildfire (13.09, 0.001); forest smoke (11.41, 0.001); chemistry transport model (11.41, 0.001); health care visits (11.41, 0.001) | redecoration (0.27); ground level ozone (0.27); acute effect (0.27); premature deaths (0.27); housing conditions (0.27) |
| 8 | 33 | 0.822 | 2019 | air pollution; childhood asthma; fossil fuel; lung neoplasms; attention deficit hyperactivity disorder \| ambient air pollution; allergic rhinitis; fossil fuel; lung neoplasms; attention deficit hyperactivity disorder | diurnal temperature range (19.27, 1.0E-4); preschool children (13.72, 0.001); child health (10.1, 0.005); ambient air pollution (9.02, 0.005); risk factor (8.35, 0.005) | long-range transport (0.22); time-series (0.22); cardiovascular health effects (0.22); particulate matter (pm2.5) (0.22); air quality standards (0.22) |
|  |  |  |  |  |  |  |
| 9 | 28 | 0.882 | 2017 | climate change; fungal-related allergic diseases; overreactivity-related diseases; flow cytometry; impacts \| impacts; algal blooms; heat; ambient temperature; health | algal blooms (14.73, 0.001); impacts (10.97, 0.001); birth weight (10.97, 0.001); grasses (7.36, 0.01); leukocytes (7.36, 0.01) | grasses (0.1); leukocytes (0.1); resilience (0.1); weeds (0.1); smartphone app (0.1) |

The table presents the main keyword clusters identified by CiteSpace, including cluster size, silhouette value, mean publication year, and cluster label generated by the log-likelihood ratio (LLR) algorithm.
